# 

**Published:** 1951-04

**Authors:** 


In Memoriam

ERNEST URQUHART BARTHOLOMEW

JOHN FREEMAN
M.D., F.R.C.S.

JOHN ALEXANDER NIXON
C.M.G., M.D., F.R.C.P.

JOHN RAYMOND EDEN SANSOM
M.B., Ch.B., B.Sc.

JAMES SWAIN
C.B., C.B.E., M.D., M.S., F.R.C.S.

JOHN ODERY SYMES
M.D.

### Obituary

### Local Medical Notes

### Appointments

**University of Bristol.—**

G. C. Lennon, Ch.M., M.R.C.O.G.—Professor of Obstetrics.

J. E. Cates, M.D., M.R.C.P.—Lecturer in Medicine.

R. E. Horton, F.R.C.S.—Lecturer in Surgery.

P. F. Redman, M.B., Ch.B., M.R.C.O.G.—Lecturer in Obstetrics.

Coralie W. Rendle-Short, M.B., Ch.B., M.R.C.O.G.—Head of Department of Obstetrics, Makerere College, Kampala, Uganda.

M. P. Embrey, M.D., B.Sc., F.R.C.S., M.R.C.O.G.—Deputy Professor, Nuffield Department of Obstetrics, University of Oxford.

C. A. Brown, M.D., D.O.M.S.—Consultant Ophthalmologist, Bristol.

F. J. Farr, M.B., D.M.R.E.—Consultant in Radiology, Dewsbury, Batley and Mirfield Hospitals.

E. J. Harries, M.D.—Consultant Pathologist, South Somerset.

S. D. Loxton, F.R.C.S.—Consultant Obstetrician and Gynaecologist, United Bristol Hospitals.

T. A. B. Mason, D.M.R.—Consultant Radiologist, West Cornwall.

T. C. St. C. Morton, *C.B.*, *O.B.E.*, M.D., F.R.C.P., D.P.H., D.P.M.—Area Pathologist, West Cornwall.

J. Winter, M.B., D.M.R.D.—Consultant Radiologist, South Somerset.

### Degrees and Diplomas

**University of Bristol.—**

M.B., Ch.B.—R. M. Barnes, Marianne A. Barry, Monica B. Bishop, K. A. Boulton, Luba Epsztejn, B. R. W. Evans, Hella R. Fluss, R. F. Harvey, M. E. Hincks, K. R. Kidd, H. Maxwell, G. A. Pilkington, J. F. Rowland, Rachel D. R. Sasieni, Mildred M. C. Shaw, D. T. I. Stuttaford, Ginette M. Tebbutt, P. A. Titterton, Patricia Vowles.

M.B., Ch.B.—*Second Examination.*—H. T. Apsimon, V. Baker, P. H. Barry, C. A. Bickford, J. B. Burn, J. T. Bush, T. H. P. Chatfield, J. R. Clamp, Catherine A. Clark, Mavis E. Coutts, G. G. Crabbe, Violet E. Dadds, R. N. Davies, F. G. Farrelly, G. N. Febry, D. W. Fortune, K. R. Gough, M. L. Harvey, Antoinette R. Joseph, F. E. S. Keiller, J. M. Laltoo, T. N. Matthews, R. G. McCormick, R. E. Midwinter (with Distinction in Anatomy), C. A. Milner, Mary E. Moore (with Distinction in Anatomy), E. J. R. Morgan, Jean D. Murray-Shirreff, G. A. Naylor, J. H. Orr, D. B. Paintin (with Distinction in Anatomy), B. K. Palmer, B. A. Poley, M. Roylance, R. K. M. Sanders, H. Savery, R. Seymour-Shove, F. G. Spear, D. M. M. Thomson, Angela C. Willis, M. R. Wills.

D.P.M.—J. A. Ardis, K. W. Aron, E. C. Turton.

B.D.S.—M. Bernstein, A. H. Chivers, Ruth A. Yearn.

L.D.S.—G. D. Everard, H. T. Jones, D. A. Pearce, W. H. N. Scawin.

### SUBSCRIBERS to the BRISTOL MEDICO-CHIRURGICAL JOURNAL

*We very much want to keep this list accurate: please notify any errors or ommissions, without delay to Dr. G. E. F. Sutton, 1 Pembroke Road, Bristol 8, All members of the Bristol Medico-Chirurgical Society are subscribers to the 'Journal'. Names in brackets are those of subscribers who have joined and resigned since* 1948.

### BRISTOL

A. W. ADAMS
R. AIDIN
J. ALDREN
G. L. ALEXANDER
G. S. ANDREWS
W. E. ARNOLD

P. BAER
W. BAIN
LILY A. BAKER
R. F. BARBOUR
R. H. BARNFIELD
C. BARTLETT
URSULA BAXTER
V. T. BAXTER
D. L. BAYLEY
(P. H. BEALES)
P. H. BEAMISH
D. H. BEATSON
H. BECK
R. J. IRVING BELL
(SHEILA BELOE)
(O. D. BERESFORD)
K. BERGIN
R. J. A. BERRY
K. BIDEN-STEELE
J. A. BIRRELL
M. BODYCOMBE
D. C. BODENHAM
F. H. BODMAN
D. BOLT
B. J. BOULTON
H. BOURNS
(R. G. BOYD)
E. G. BRADBEER
R. G. BRENNEN
WINIFRED P. BRINCKMAN
BRISTOL GENERAL HOSPITAL
BRISTOL ROYAL INFIRMARY
B.R.I. STUDENTS' COMMON ROOM
R. B. BRITTON
R. J. BROCKLEHURST
(J. K. BROWNRIGG)
C. A. BROWN
N. BROWN
N. F. W. BRUETON
EUNICE BRYANT
MARGARET H. BUSTON
T. J. BUTLER

F. J. CAIRNS
D. CAMERON
MARGARET CAMERON
W. CAMERON
A. M. G. CAMPBELL
H. CAPLAN
W. M. CAPPER
T. N. CARAYANOPOULOS
R. S. CAREY
T. H. CARLETON
T. G. CARMICHAEL
W. H. K. CARPENTER
R. N. CATES
J. H. CHALLENGER
E. R. CHAMBERS
W. A. CHAPMAN
(K. M. CHIPPERFIELD)
L. E. CLAREMONT
R. C. CLARKE
F. CLIFTON
(J. A. COCHRANE)
ELIZABETH M. COOKE
N. COOKE
R. V. COOKE
C. CORFIELD
BERYL CORNER
J. A. COSH
W. L. COSSHAM
H. H. COULTER
R. M. COURTNEY
BEATRICE COXON
ANNE A. CRAIG
N. S. CRAIG
R. A. CRAIG
G. I. CULANK
S. CURWEN

A. H. DALE
A. I. DARLING
A. A. K. DATTA
S. S. DATTA
D. H. DAVIES
H. J. DAVIES
N. DELANEY
MARGOT DICKSON
C. DIX
MARY DIXON
A. A. DOWLING
J. R. DUERDEN
MARJORIE DUNSTER
HILDA R. DUTTON

J. ELLIOTT
W. H. G. ELLIOTT
W. T. ELLIS
(H. ELLIS)
C. EVANS
G. M. EVANS
J. EVANS
A. L. EYRE-BROOK
E. M. EYRE-BROOK

H. D. FAIRMAN
P. FARMER
G. R. FELLS
R. R. FELLS
G. L. FENELEY
H. P. M. FINZEL
H. J. W. FISHER
G. M. FITZGIBBON
A. A. G. FLEMMING
D. H. FORSTER
G. L. FOSS
A. D. FRASER
C. JEAN FRASER
SHEILA FRASER

A. H. GALE
R. R. GARDEN
E. B. GARLAND
E. T. GARTHWAITE
B. F. GIBSON
W. A. GILLESPIE
H. M. GOLDING
A. P. GORHAM
(J. L. T. GRAHAM)
J. M. GRAHAM
J. GRAYSON

H. E. HARRIS
GRETA HARTLEY
F. HECTOR
A. G. HERON
T. F. HEWER
M. HEWITT
M. P. HICKEY
S. W. HINDS
WINIFRED HISCOCK
W. A. L. HOLLAND
R. E. HORTON
H. HOWELL
LYDIA M. HOUGHTON
H. HOWELL
(T. E. HOWELL)
T. G. F. HUDSON
W. HUGHES
F. S. HUNTER

ANNA ILES
A. E. ILES
B. ISSERLIN
J. A. JAMES
P. JARDINE
F. G. JENKINS
R. G. JOHNSON
D. M. JONES
P. C. JOSCELYNE

G. C. KELLY
T. S. B. KELLY
N. KEMM
S. H. KINGSTON
J. KNIGHT
H. G. KYLE

E. J. LACE
M. E. LACE
G. E. P. LEE
LOIS M. LEITCH
M. LENNARD
G. C. LENNON
A. LEVY
F. J. LEWIS
C. LINTON
S. D. LOXTON
J. E. LUCAS
K. LUCAS
J. J. S. LUCAS
R. P. LUCAS

B. E. McCONNELL
M. P. McCORMICK
P. T. T. MACDONALD
G. K. MCGOWAN
D. MACKAY
A. M. MACLACHLAN
CATHERINE J. MACLAREN
A. C. McPHERSON
J. MACRAE
C. V. M. MAHOOD
S. F. MARWOOD
J. MASON
J. J. MASON
S. MASON
D. MAUNSELL
J. D. MEDHURST
J. MIDDLEMISS
A. MILLER
T. MILLING
F. G. MOGG
S. L. MOHAN
R. H. MOORE
C. C. MORGANS
D. F. MURPHY
Q. ST. L. MYLES

A. V. NEALE
A. S. NETHERCOTT
M. A. NICHOLSON
DOREEN NIXON
J. I. NOBLE
R. M. NORMAN
(C. T. NORRIS)
G. L. ORMEROD
H. J. ORR EWING
B. F. ORTON
R. W. ORTON
L. W. OXENHAM
R. H. OWEN

K. PAGE
A. G. PALIN
P. W. J. PARKES
T. J. PARKINSON
T. PARR
R. H. PARRY
R. G. PAUL
J. H. PEACOCK
RUTH PERKS
C. B. PERRY
D. G. PHILLIPS

P. PHILLIPS
A. L. PIRRIE
D. V. PLEASANT
J. A. POCOCK
A. V. POOTS
MABEL F. POTTER
C. H. G. PRICE
K. H. PRIDIE
MRS. K. H. PRIDIE
S. L. PUGMIRE
H. D. PYKE

P. C. RANKIN
C. P. REEVE
F. B. RICHARDS
J. RIDGWAY
A. T. ROBERTS
J. A. F. ROBERTS
J. A. L. ROBERTS
D. ROBERTSON
L. G. ROBERTSON
R. ROLFE
A. R. ROWE
G. DE M. RUDOLF
E. A. RUHEMANN
(MEGAN RYAN)

O. E. L. SAMPSON
E. SAPHIER
G. R. SCARFF
H. L. SHEPHERD
A. R. SHORT
S. SILVEY
D. SIMPSON
T. E. N. SIMPSON
P. S. SINCLAIR
J. SLUGLETT
J. SMITH
H. J. D. SMYTHE
J. W. E. SNAWDON
D. K. SOLTAU
H. K. V. SOLTAU
H. SOSNOWICK
T. R. STEEN
(G. STEINER)
R. J. STEPHEN
I. McD. G. STEWART
J. M. STOBO
A. J. STRUTHERS
G. STRUTHERS
MRS. G. STRUTHERS
G. E. F. SUTTON

E. A. TARLETON
D. G. C. TASKER
(W. F. T. TATLOW)
A. L. TAYLOR
J. T. C. TAYLOR
MARY TAYLOR
A. T. TODD
VICTORIA S. TRYON
R. C. TUDWAY

N. S. B. VINTER

MONICA WAITE
R. P. WARIN
C. H. WALKER
T. B. WANSBROUGH
E. WATSON-WILLIAMS
E. J. WATSON-WILLIAMS
A. WEEKS
MARO WELLS
PAMELA WESTHEAD
R. R. WETHERED

J. H. WHITTLES
R. G. WILBOND
I. WILLIAMS
K. L. WILSON
H. WOHLFELD
DOROTHY WOODMAN
G. E. WOODS
W. WOOLLEY
R. WOOLMER

J. M. YOFFEY

### BERKSHIRE

*Bracknell*

J. J. KEMPTON

### CHESHIRE

*Bowden*

G. E. ARCHER

### CORNWALL

FOWEY HOSPITAL
ST. AUSTELL HOSPITAL

*Penzance*

R. W. PAYNE

*Redruth*

T. S. WILSON

*Truro*

C. T. ANDREWS

### CUMBERLAND

*St. Bees*

F. V. JACQUES

### DURHAM

*Barnard Castle*

N. C. COOMBS

### DEVONSHIRE

*Barnstaple*

(K. G. W. SAUNDERS)

*Crediton*

MARGARET C. N. JACKSON
L. N. JACKSON

*Ivybridge*

R. W. L. WALKER

*Paignton*

(J. F. BURDON)
(P. M. GARRY)

*Bideford*

S. C. WAKE

*Dawlish*

(W. P. WIPPELL)

*Newton Abbot*

D. CROMIE
G. M. TANNER

*Plymouth*

G. BALL
C. R. CROFT

*Teignmouth*

BERTHA M. MULES

*Buckfastleigh*

S. R. WILLIAMS

*Exeter*

M. I. GOVIER
R. HADDEN
C. M. SEWARD

*Torquay*

W. S. BAXTER
W. ETHERINGTON-WILSON
P. C. GIBSON
R. A. LATTEY
M. LEES
P. A. MCCALLUM
R. SAMPSON
(B. VENN-DUNN)

### ESSEX

*Ilford*

(WINIFRED HILL)

### GLOUCESTERSHIRE

*Almondsbury*
B. P. GRIFFIN

*Charfield*
W. J. GALL

*Cheltenham*
H. S. ALLEN
E. D. D. DAVIES

*Chipping Sodbury*
A. H. CASSON
G. S. MUNDY
J. NAISH

*Cirencester*
D. G. COSSHAM

*Dursley*
(W. J. D. COWPER)
B. W. D. FAYLE
D. FRASER

*Frenchay*
R. BELSEY
K. H. GASKELL
E. L. HUTTON
EVELYN MAWSON

*Gloucester*
C. DE W. GIBB
H. A. HAMILTON

*Hambrook*
F. W. CROSSMAN
H. J. O'SULLIVAN

*Lechlade*
DR. FITZGERALD

*Mangotsfield*
J. M. CORMACK

*Pilning*
E. P. K. RUSHTON

*Pucklechurch*
W. W. PRATT

*St. Briavels*
J. ESKELL

*Stonehouse*
STANDISH HOUSE SANATORIUM

*Thornbury*
D. HENDERSON
D. C. PROWSE

*Warmley*
J. C. K. CHILCOTT

*Wick*
A. L. SMALLWOOD

*Wotton-under-Edge*
P. WILLIAMS

*Winterbourne*
J. E. CATES

### HAMPSHIRE

*Basingstoke*
(MERCIA J. BRADLEY)

*Romsey*
T. P. LALONDE

*Cowes, I.O.W.*

J. A. HOOKER

### KENT

*Dartford*
E. H. RAINER

### LANCS.

*Liverpool*
MRS. J. J. BALL
C. KOMOCKI

### LONDON

A. F. ALFORD
R. F. BOLT
P. JACOBS
E. F. KING
WINIFRED LEWIS
M. O'CONNOR
SIR J. H. PARSONS

### MIDDLESEX

*Harefield*
(W. D. G. COOPER)

### NORTHUMBERLAND

*Newcastle-on-Tyne*
B. CAMERON
G. A. SMART

### SOMERSET

*Axbridge*
J. BLAKE

*Bridgwater*
P. LEGAT

*Clevedon*
A. G. W. BRANCH
ELIZABETH CASSON
L. C. HYLTON
W. H. HYLTON
P. K. JENKINS
G. MACLEOD
I. A. MCLEOD
H. OAKES

*Felton*
J. V. SPARKS

*Keynsham*
J. G. FIELD
N. GERRISH

*Nailsea*
NAOMI J. BOWN
W. A. GORNALL
R. F. WHITE
J. WILLOUGHBY

*Portishead*
(G. B. BUSH)
W. A. JACKMAN
J. L. PARDOE
MRS. M. M. PARDOE

*Street*
J. H. C. EGLINTON

*Taunton*
G. G. ALLAN
J. BENN
J. A. BOYCOTT
M. MCALLEY
J. R. NICHOLSON-LAILEY
H. PEARSE
T. L. H. SHORE
C. E. M. WARE

*Barrow Gurney*
(G. GARMANY)
R. HEMPHILL

*Chew Magna*
F. W. T. HUGHES

*Chew Stoke*
C. H. PAULI
M. A. PAULI

*Clapton-in-Gordano*
H. CHITTY

*Freshford*
C. N. VAISEY

*Langport*
A. SPOOR

*Limpley Stoke*
W. H. HALL

*Norton Fitzwarren*
TONE VALE HOSPITAL

*Shepton Mallet*
G. W. R. BISHOP
WINIFRED BYNT

*Stoke-under-Ham*
J. L. BRIMBLECOMBE

*Timsbury*
B. A. CROOK

*West Harptree*
G. M. PEARSON

*Wrington*
D. R. GRAY
R. MILNES WALKER

*Yatton*
W. V. WOOD

*Bath*
A. J. P. ALEXANDER
J. APLEY
J. BASTOW
A. D. BATEMAN
A. E. BURTON
D. J. COOPER
MAUD FORRESTER-BROWN
H. L. FULLER
S. GLASER
L. C. HILL
H. L. HOFFMAN
G. D. KERSLEY
C. E. KINDERSLEY
F. LACE
A. LEECH-WILKINSON
F. D. MURPHY
D. E. OLIFF
T. PRICE
D. PUGH
C. P. SAMES
J. M. SANSON
(G. D. STEVEN)

*Long Ashton*
J. E. BEVISS
K. M. COSSENS
M. P. EMBREY
R. MAGGS
A. V. NEALE

*Paulton*
C. L. O. MIALL

*Somerton*
C. D. INGLE

*Weston-super-Mare*
W. R. BLACK
D. O. CLARK
J. N. HEALES
A. B. KETTLE
E. LITTLE
K. C. MALLEN
P. S. MARTIN
W. J. PETTY
H. F. POWELL
A. T. ROWLEY
K. S. SINCLAIR
MARY SOMERS
R. WARREN

### STAFFORDSHIRE

*Wolverhampton*

P. C. PHELPS

*Lichfield*

RUTH M. ROCKE

### SUFFOLK

*Ipswich*

(G. F. LANGLEY)

### SURREY

*Purley*

F. C. COLLINGWOOD

### SUSSEX

*Eastbourne*

J. B. ADAMS

*Worthing*

(J. R. GIBBS)

### WARWICKSHIRE

*Birmingham*

(J. HENDERSON)
DOROTHY M. SHOTTON

*Coventry*

JOYCE W. BROWN

### WILTSHIRE

*Bradford-on-Avon*

I. F. MCMATH

*Devizes*

J. M. C. SPEER

*Marshfield*

H. J. EASTES
ZETA EASTES

*Stratton*

J. LOWE

*Upavon*

L. C. HOLLAND

### WORCESTERSHIRE

*Kidderminster*

C. P. PORTER

### YORKSHIRE

*Bradford*

H. E. C. BENTLEY

*Bridlington*

S. A. SWANSON

*Sheffield*

(J. L. EMERY)

### WALES

### BRECONSHIRE

*Sennybridge*

N. E. MELLING

### GLAMORGANSHIRE

*Cardiff*

J. H. BULLEID
C. A. GRIFFITHS
SIR EWEN J. MACLEAN
R. D. OWEN

### IRELAND

### ANTRIM

*Belfast*

R. H. TRINICK

### SCOTLAND

*Edinburgh*

A. L. TAYLOR
J. THIN

### AFRICA

ZULULAND
*Ngutu*
WINIFRED N. TRIBE

UGANDA
*Kampala*
H. J. CROOT
J. N. P. DAVIES

ZANZIBAR
R. J. K. TALLACK

### AMERICA

*Trinidad, B.W.I.*
J. E. BROWN

### AUSTRALIA

VICTORIA
*Melbourne*
(C. W. WEDDELL)

### INDIA

HYDERABAD
*Karim Nagar*
DOROTHY TRIPP

### SWEDEN

*Stockholm*
KAROLINSKA INSTITUTETS BIBLIOTECK

